# Supplementary material for: Cricket ball in the right heart: A case report of right atrial myxoma
Source: Ann Med Surg (Lond). 2019 Oct 17;48:39–42. doi: 10.1016/j.amsu.2019.10.015 (PMC6820116; doi:10.1016/j.amsu.2019.10.015)
Supplement: Multimedia component 1 [file mmc1.docx]

| **SCARE Checklist** | | | |
| --- | --- | --- | --- |
| **Topic** | **Item** | **Checklist item description** | **Page Number** |
| **Title** | **1** | **CRICKET BALL IN THE RIGHT HEART** | 1 |
| **Key Words** | **2** | Myxoma, 2-D Echo, Right atrium. | 1 |
| **Abstract** | **3a** | **Introduction:** Myxomas are the most common type of cardiac benign tumors and most of them are located in the left atrium, followed by the right atrium. The majority of Myxomas is located in the left atrium and has a variable clinical presentation. Myxomas affect patients within a wide age range (15 to 80 years), and the average age is approximately 50 years. There is a female predominance in the sporadic form. Myxomas are usually pedunculated, solitary, and sporadic but may be associated with familial autosomal dominant syndromes. | 1 |
|  | **3b** | **Case Presentation:** We report a 38-year-old female presented with large myxoma in the right atrium and atypical presentation and successfully underwent surgical excision of right atrial myxoma. |  |
|  | **3c** | The patient was asymptomatic on 6 months follow up. |  |
|  | **3d** | **Conclusion:** Cardiac myxomas are the most frequent finding among primary cardiac tumors. Clinical manifestations depend on the involvement in valvular obstruction, distant arterial embolisms, or nonspecific, constitutional symptoms. Transoesophageal Echocardiography is the cornerstone for diagnosis of atrial myxoma. Cardiac myxomas should be managed with surgical resection. |  |
| **Introduction** | **4** | Cardiac tumors are divided into primary and secondary tumors. Primary cardiac tumors are very rare, with an incidence of 0.001% to 0.03%[1]. Secondary involvement of the heart by extracardiac tumors is 20 to 40 times more common than by primary cardiac tumors [2]. More than 80% of primary cardiac tumors are benign, and myxoma is the most common benign cardiac tumors, constitutes approximately about 50% of all benign cardiac tumors in adults [3]. About 80% to 90% of Myxomas located is in the left atrium [4]. Right atrial myxoma accounts for only 15% to 20% of all cardiac Myxomas. And less than 2.5% are biatrial [5]. cardiac myxomas are rarely found in the ventricles, valves. Myxomas most commonly presents in adults 3^rd^ to 6^th-^decade of life, but it can occur in all age groups ranging from 1 to 83 years [6]. With a female-to-male ratio of approximately 3:1 [7]. Most myxomas occur sporadically but may be familiar. Familial tumors are more likely to be multiple, recurrent and right-sided compared to sporadic myxomas. | 2 |
| **Patient Information** | **5a** | We report a case of 40-year-old female patient, presented with chest pain, epigastric abdominal pain, vomiting for 3 days, easy fatigability since 1 yr, intermittent episodes of presyncope since 1 year. No constitutional disturbances such as fever, weight loss, skin rash, myalgia or arthralgia. | **3** |
|  | **5b** | Cardiovascular system examination was normal with no clinically detectable murmurs. |  |
|  | **5c** | No past history of surgery. |  |
|  | **5d** | No Drug history, No family history not a smoker or alcoholic. |  |
| **Clinical Findings** | **6** | ECG showed sinus rhythm, incomplete RBBB with T inversion in leads III and avF. Chest X-Ray revealed normal. Transthoracic Echocardiography revealed a large mass in the right atrium, attached to the interatrial septum. Trans-Esophageal Echocardiography was done to clearly delineate the mass, which confirmed the attachment of RA mass to the interatrial septum in the region of fossa ovalis, with no extension into inferior vena cava, measuring 5.0 x 4.0 cm [Fig 2A & 2B]. Ultrasound abdomen done was normal with no renal mass. Blood investigations done were normal. CT Angio showing 46x40x40 sized hypo density seen in right atrium minimal extension into IVC atrial junction [Fig 1]. The patient was suspected to have RA myxoma. The patient underwent open surgical resection of the RA mass with no pre-operative or post-operative complications [Fig 3]. Gross macroscopic examination showed a nearly spherical gelatinous mass of the size of a cricket ball measuring around 4 cm in diameter with a smooth surface and elastic consistency. Cut section of the mass showed a dark red hemorrhagic core surrounded by pale-gray surface layer. Histo-pathological examination confirmed the diagnosis of cardiac myxoma. Microscopic showing tumor displaying myxoid change and stellate cells having bland nuclei arranged in reticular meshwork. Large areas of hemorrhage are noted, dark-colored pigmentation and a few pigment-laden macrophages are present [Fig 5A & 5B]. The patient was asymptomatic on 6 months follow up. 2D Echo revealed normal [Fig 6].  Our case is important because of the atypical size and location of cardiac myxoma in the right atrium. | 3 |
| **Timeline** | **7** | Transthoracic Echocardiography revealed a large mass in the right atrium, attached to the interatrial septum. Trans-Esophageal Echocardiography was done to clearly delineate the mass, which confirmed the attachment of RA mass to the interatrial septum in the region of fossa ovalis, with no extension into inferior vena cava, measuring 5.0 x 4.0 cm. The patient was suspected to have RA myxoma. The patient underwent open surgical resection of the RA mass with no pre-operative or post-operative complications. | 3 |
| **Diagnostic Assessment** | **8a** | Physical exam, Laboratory testing, Chest X-Ray, ECG, Transthoracic Echocardiography, Trans-Esophageal Echocardiography, CT Aortagram. | 3 |
|  | **8b** | N/A |  |
|  | **8c** | N/A |  |
|  | **8d** | N/A |  |
| **Therapeutic Intervention** | **9a** | N/A | 4 |
|  | **9b** | N/A |  |
|  | **9c** | N/A |  |
|  | **9d** | N/A |  |
|  | **9e** | N/A |  |
|  | **9f** | N/A |  |
| **Follow-up and**  **Outcomes** | **10a** | Patient was on regular follow up. |  |
|  | **10b** | N/A |  |
|  | **10c** | N/A |  |
|  | **10d** | N/A |  |
| **Discussion** | **11a** | Right atrial myxoma accounts for only 15% to 20% of all cardiac myxomas. It is usually found in the interatrial septum, RA myxomas usually originate in the fossa ovalis or base of the interatrial septum, but in our case, the myxoma was attached to interatrial septum, prolapsing through the tricuspid valve into the right ventricle. Myxomas are usually polypoid and pedunculated tumors (approximately 83% of cases). Atypical locations and multiple myxomas occur most frequently in cases of familial myxoma. Myxomas are usually polyploidy and pedunculated. Mobile myxomas often exacerbate shortness of breath when the patient assumes a particular posture, but we could detect no such relationship. The motion of the tumor can damage the atrioventricular valve and rupture the chordate. Our patient had a Gross a globular reddish brown soft tissue mass measuring 5X4X4cm. |  |
|  | **11b** |  |  |
|  | **11c** |  |  |
|  | **11d** |  |  |
| **Patient Perspective** | **12** | Procedure details are explained to Patient. |  |
| **Informed Consent** | **13** | Patient consent was obtained. |  |
| **Additional Information** | **14** | Conflicts of Interest: Nothing  Sources of funding: None  Ethical committee approval: Obtained. | 5 |
